# Complete Genome Sequences for Two Strains of a Novel Fastidious, Partially Acid-Fast, Gram-Positive *Corynebacterineae* Bacterium, Derived from Human Clinical Samples

**DOI:** 10.1128/genomeA.01462-15

**Published:** 2015-12-10

**Authors:** Ainsley C. Nicholson, Melissa Bell, Ben W. Humrighouse, John R. McQuiston

**Affiliations:** Special Bacteriology Reference Laboratory, Bacterial Special Pathogens Branch, Division of High Consequence Pathogens and Pathology, Centers for Disease Control and Prevention, Atlanta, Georgia, USA

## Abstract

Here we report the complete genome sequences of two strains of the novel fastidious, partially acid-fast, Gram-positive bacillus “*Lawsonella clevelandensis*” (proposed). Their clinical relevance and unusual growth characteristics make them intriguing candidates for whole-genome sequencing.

## GENOME ANNOUNCEMENT

The Special Bacteriology Reference Laboratory receives unusual and difficult-to-identify bacterial strains derived from clinical specimens from throughout the United States. Two strains of a novel fastidious, partially acid-fast, Gram-positive *Corynebacterineae* bacterium (X1036^T^ and X1698) were obtained from human abscesses, as previously described (1). The strains grow best in an atmosphere generated by a GasPak in an anaerobe jar (≤1% O_2_), including the stringent environment of an anaerobe chamber. Limited growth is achieved under the conditions created by a CampyPak (6 to 16% O_2_) and little to no growth in ambient air. (GasPak and CampyPak are products of Becton, Dickinson and Company, USA.)

Genomic DNA was extracted from cells grown on CDC anaerobic blood agar (BD) in a GasPak for 2 to 3 weeks. Cells were harvested and gDNA was prepared using the Epicentre Metagenomic DNA isolation kit for water (Illumina, USA). Genome libraries were prepared using the NEB Ultra DNA library prep kit (New England Biolabs, USA), according to the manufacturer’s instructions, on a PerkinElmer Sciclone NGS robot. Sequence reads were 100-bp × 100-bp paired-end reads on a HiSeq2500 operating in rapid mode; 12,496,108 (X1036^T^) and 12,804,044 (X1698) sequence reads were generated. Reads were trimmed based on quality (limit = 0.05), and assembled using the De Bruijn graph method of *de novo* assembly provided by CLC genomics workbench version 7.04, with default parameters.

AflIII (both strains) and HinDII (X1698 only) optical maps were analyzed using Argus MapSolver software (OpGen, USA) to order and orient contigs with respect to each other. Gaps were closed using BioEdit (2) based on alignments of reads: reads corresponding to the ends of each contig were located, sorted, and aligned using JMP version 10 (SAS Institute Inc., USA), and then visualized using BioEdit. This process was iterated as needed to close gaps that could not be closed using the De Brujin graph method.

Both genomes were automatically annotated by the PGAP pathway at NCBI, and the resulting open reading frames were compared with RAST (3) and KEGG annotations (4). Their ability to perform aerobic respiration is evidenced by the presence of both cytochrome *d* complex and all subunits of proton-translocating NADH-dehydrogenase genes (nuoA-N).

### Nucleotide sequence accession numbers.

The complete genome sequences of X1036^T^ and X1698 have been deposited at GenBank under the accession numbers CP009312 and CP012390, respectively. Both are part of the BioProject PRJNA256353.
